# A New PCR-Based Approach Indicates the Range of *Clonorchis sinensis* Now Extends to Central Thailand

**DOI:** 10.1371/journal.pntd.0000367

**Published:** 2009-01-20

**Authors:** Rebecca J. Traub, Julie Macaranas, Mathirut Mungthin, Saovanee Leelayoova, Thomas Cribb, K. Darwin Murrell, R. C. Andrew Thompson

**Affiliations:** 1 School of Veterinary Science, University of Queensland, St. Lucia, Queensland, Australia; 2 Department of Parasitology, Phramongkutklao College of Medicine, Bangkok, Thailand; 3 School of Molecular and Microbial Sciences and Centre for Marine Studies, University of Queensland, St Lucia, Queensland, Australia; 4 Department of Veterinary Pathobiology, Faculty of Life Sciences, University of Copenhagen, Fredericksberg, Denmark; 5 World Health Organization Collaborating Centre for the Molecular Epidemiology of Parasitic Infections, School of Veterinary and Biomedical Sciences, Murdoch University, Murdoch, Western Australia, Australia; Khon Kaen University, Thailand

## Abstract

Differentiation of the fish-borne trematodes belonging to the Opisthorchiidae, Heterophyidae and Lecithodendriidae is important from a clinical and epidemiological perspective, yet it is impossible to do using conventional coprological techniques, as the eggs are morphologically similar. Epidemiological investigation therefore currently relies on morphological examination of adult worms following expulsion chemotherapy. A PCR test capable of amplifying a segment of the internal transcribed spacer region of ribosomal DNA for the opisthorchiid and heterophyid flukes eggs taken directly from faeces was developed and evaluated in a rural community in central Thailand. The lowest quantity of DNA that could be amplified from individual adults of *Opisthorchis viverrini*, *Clonorchis sinensis* and *Haplorchis taichui* was estimated at 0.6 pg, 0.8 pg and 3 pg, respectively. The PCR was capable of detecting mixed infection with the aforementioned species of flukes under experimental conditions. A total of 11.6% of individuals in rural communities in Sanamchaikaet district, central Thailand, were positive for ‘*Opisthorchis*-like’ eggs in their faeces using conventional parasitological detection techniques. In comparison to microscopy, the PCR yielded a sensitivity and specificity of 71.0% and 76.7%, respectively. Analysis of the microscopy-positive PCR products revealed 64% and 23% of individuals to be infected with *O. viverrini* and *C. sinensis*, respectively. The remaining 13% (three individuals) were identified as eggs of Didymozoidae, presumably being passed mechanically in the faeces following the ingestion of infected fishes. An immediate finding of this study is the identification and first report of a *C. sinensis*–endemic community in central Thailand. This extends the known range of this liver fluke in Southeast Asia. The PCR developed herein provides an important tool for the specific identification of liver and intestinal fluke species for future epidemiological surveys.

## Author Summary

It is estimated that approximately 17 million people are currently infected with fish-borne flukes worldwide. The fish-borne liver flukes *Opisthrochis viverrini* and *Clonorchis sinensis* cause hepatic and biliary disease in humans. The minute intestinal flukes are widely distributed in southeast Asia and are increasingly recognised as an emerging pathogen associated with diarrhoea and gastritis. The most significant finding of this study is the discovery and first report of a *C. sinensis*–endemic community in Thailand. This finding was aided by the development and application of a new PCR-based technique capable of specifically detecting and characterising *O. viverrini*, *C. sinensis* and the minute intestinal flukes, directly from eggs in faeces. Since the eggs are morphologically similar, the fish-borne flukes cannot be differentiated on basis of microscopic examination of stool. This publication also questions the presumption that the distribution of fish-borne liver fluke species in Asia closely parallels the distribution of the snail intermediate hosts. The PCR provides a useful diagnostic tool for further large-scale epidemiological surveys to be carried out in Southeast Asia, which will shed further light on the distribution of these liver flukes in human and snail intermediate hosts with the advantage that targets for more arduous anthelmintic flushing confirmations can be carried out.

## Introduction

It is estimated that approximately 17 million people are currently infected with fish-borne trematodes worldwide [1]. In Asia, *Opisthorchis viverrini* is known to occur in Thailand, Laos, Cambodia and southern Vietnam and *Clonorchis sinensis* in Korea, China, Taiwan and northern Vietnam [2],[3].

Liver fluke infection in Thailand is unevenly distributed with a highly endemic focus of infection in the northeast region [4]. Previous parasite surveys have mostly focussed on these communities and frequently found infection with *O. viverrini* mixed with minute intestinal flukes of the Heterophyidae and Lecithodendriidae [5],[6]. The heterophyids *Haplorchis taichui* and less frequently *H. pumilio* are the most common minute intestinal flukes recovered.

Microscopic examination of faecal samples for the presence of eggs using the formalin-ether concentration technique (FECT) is currently considered the most sensitive and reliable method for screening liver and intestinal flukes and is therefore the most widely employed technique for fluke parasite surveys [7]. This technique is limited by its capacity to differentiate between the Opisthorchiidae, Heterophyidae and Lecithodendriidae, which have similar egg morphologies. Eggs can therefore only be characterised as ‘*Opisthorchis*/*Clonorchis*- like’ [5],[6], but no further. A definitive diagnosis to species level requires morphological identification of adult flukes following expulsion chemotherapy [8],[9]. The ability to differentiate the species of liver and minute intestinal flukes is important from both a clinical and epidemiological perspective. Heavy infections with the minute intestinal flukes are associated with diarrhoea, mucus-rich faeces, dyspepsia, nausea and vomiting [10], whereas infections with the liver flukes result in mostly biliary and hepatic disease. The frequency and types of pathology and clinical disease among *C. sinensis* and *O. viverrini* also seem to differ [7]. For example, cholelithiasis is one of the more serious complications of clonorchiasis, but a rare complication of opisthorchiasis. Although both flukes are implicated as predisposing factors for cholangiocarcinoma, this is more frequent with *O. viverrini*. From an epidemiological perspective, *C. sinensis* has a wider definitive host range than *O. viverrini* [11],[12] which makes control more challenging.

To overcome the diagnostic limitations associated with conventional parasitological methods, a number of PCR-based techniques capable of amplifying species of flukes directly from eggs in faeces have been developed [13]–[16]. An *O. viverrini*-specific PCR test capable of detecting *O. viverrini* eggs directly from human faeces was shown to have an analytical sensitivity of 100%, 68.2% and 50% compared to the Stoll's egg count containing >1000, 200 to 1000 and <200 eggs per g of faeces respectively and an analytical sensitivity of 97.8% under experimental conditions [16]. This PCR assay proved less reliable however, once evaluated under field conditions with an overall diagnostic sensitivity of 45% compared to the FECT [17]. PCR tests based on amplification of the mitochondrial gene for the identification and discrimination of *C. sinensis* and *O. viverrini* [13] and *C. sinensis*, *O. viverrini* and *H. taichui* [15] have been developed and shown to be analytically sensitive under experimental conditions. Amplicons for the targeted fluke species could be obtained in reactions containing 0.78 ng of genomic DNA [13] and 10^−4^ ng of genomic DNA [15], however the assays have yet to be evaluated and compared to conventional parasitological methods in the field.

Here we developed a PCR test capable of specifically amplifying a segment of the internal transcribed spacer (ITS-2) region of ribosomal DNA (rDNA) from opisthorchiid and heterophyid flukes directly from eggs in faeces. The ITS-2 has successfully discriminated species from many digenean families and has become the default region of choice for distinguishing species of trematodes [18]. This PCR test is evaluated in terms of both analytical and diagnostic sensitivity and specificity against conventional parasitological methods in a community endemic for liver fluke infection in central Thailand. This study is also the first to demonstrate the occurrence of a *C. sinensis* endemic community in Thailand.

## Methods

### Stool sampling and parasitological methodology

A rural community consisting of a total population of approximately 5465 people in Nayao village, Sanamchaikaet District, Chachoengsao Province, 150 km east of Bangkok was chosen for this cross-sectional study. The area lies in a low basin of land which is suitable for cultivation of rice which provides the principle income for the province. The dietary habit of eating raw and fermented fish dishes such as ‘*koi pla*’, ‘*pla som*’ and ‘*pla ra*’ from fish sourced at local ponds is popular among residents of this community. Houses were chosen at random and household members informed of the study by medical students from the Phramongkutklao College of Medicine. After signing human ethics consent forms, single stool samples were collected from a total of 335 individuals of all ages, gender and backgrounds during a 10-day period in mid November 2004. Participants found positive for gastrointestinal parasites received free anthelmintic treatment from the medical doctors on the research team in order to increase compliance. All samples were qualitatively evaluated and run in parallel for the presence of *Opisthorchis*-like eggs using a direct faecal smear (DFS), Kato Katz (KK) technique and the FECT by experienced parasitology technicians from the Phramongkutklao College of Medicine in the field. Any remaining faecal material was fixed in 20% dimethylsulfoxide (DMSO) saturated with salt for transport to the University of Queensland for molecular testing.

A single individual found positive for ‘*Opisthorchis*-like’ eggs in their faeces was treated with a single dose of praziquantel (40 mg per kg) and was then given 30 g magnesium sulfate with as much water as possible to facilitate expulsion of adult flukes. Whole diarrhoetic stools were collected and washed several times before isolating the adult flukes [9]. Adult flukes that had been expelled by this individual were fixed in 70% ethanol for molecular and morphological identification at the University of Queensland. Morphological identification was performed by staining the adult fluke with haematoxylin, dehydrating it in alcohol and clearing it in methyl salicylate before mounting in Canada balsam.

This study was approved by the Murdoch University Human and Animal Ethics Committees of Western Australia and the Ethical Committee, the Medical Department Royal Thai Army.

### DNA extraction of adult flukes and ‘*Opisthorchis*-like’ eggs in faeces

Adult flukes of *O. viverrini*, *C. sinensis* and *H. taichui* were extracted using the Qiagen DNeasy Blood and Tissue Kit according to manufacturer's instructions. Those faecal samples found microscopically positive for ‘*Opisthrochis*-like’ eggs using at least one parasitological test and where sufficient quantities of stool remained, were subjected to DNA extraction and PCR (n = 31). In addition, 30 faecal samples negative for ‘*Opisthorchis*-like’ eggs by microscopy were also randomly selected and subjected to DNA extraction and PCR. All PCR reactions were conducted by a single experienced molecular biologist that was blind to the results of the parasitological test results for each sample. It was observed that subjecting ‘*Opisthorchis*-like’ eggs purified by a saturated salt and glucose gradient to freezing in liquid nitrogen followed by thawing them at 98–100°C resulted in the eggs ‘disintegrating’ to release genomic DNA. Two hundred milligrams of faeces were suspended in 1.4 ml ATL tissue lysis buffer (Qiagen, Hilden, Germany) and this suspension subjected to 5 cycles of freezing-thawing at liquid nitrogen temperatures. DNA was then isolated from the supernatant using the QIAamp DNA Mini Stool Kit according to manufacturer's instructions. Final elutions of DNA were made in 50 µl of elution buffer instead of 200 µl as recommended by the manufacturer.

### PCR Assays and DNA sequencing

Sequences of the ITS-2 region of *C. sinensis*, *O. viverrini*, *O. felineus*, *H. taichui*, *H. pumilio* and *Centrocestus sp.* (GenBank accession nos. EF688144, EF688143, AY584735, DQ513403, DQ513405, AY245705, AY245706, AY245699) were aligned using Clustal W (http://align.genome.jp/) and the primer pair: RTFlukeFa 5′CTTGAACGCACATTGCGGCC-3′ and RTFlukeRa 5′-CACGTTTGAGCCGAGGTCAG-3′ were designed to amplify a 375 bp, 381 bp and 526 bp region of *O. viverrini*, *C. sinensis* and *H. taichui*, respectively, The PCR primers, were also designed with the potential to amplify other species of opisthorchiid and heterophyid flukes. The PCR assay was carried out in a volume of 20 µl containing 1×PCR buffer from Qiagen (Tris-HCl, KCl, (NH4)_2_SO4, 1.5 mM MgCl2; pH 8.7) additional MgCl_2_ to give a final 2.0 mM concentration, 200 µM of each dNTP, 0.25 µM of each primer, and 1 unit Hot Star Taq DNA polymerase (Qiagen). The PCR cycle consisted of an initial stage: 94°C for 15 min, 60°C for 1 min and 72°C for 2 min followed by 35 cycles of 94°C for 30 sec, 60°C for 30 sec, 72°C for 30 sec, a final extension at 72°C for 7 min and a holding temperature of 12°C.

PCR products were run on 1.5% agarose in 1×TAE buffer at 150V in a Biorad electrophoresis system and were purified using Qiagen spin columns (Qiagen) prior to sequencing. Where a multi-banded product was obtained, target bands were excised, frozen and cleaned up with a Quantum Prep Freeze ‘N Squeeze DNA Gel Extraction spin column (Biorad) or a Qiaquick Gel Extraction kit (Qiagen). Sequencing was done using an ABI 3130xl Genetic Analyzer (Applied Biosystems) using Big Dye 3.0 chemistry, after which sequences were edited and assembled using Chromas Pro (Technelysium Pty Ltd).

Titration experiments were conducted to determine the analytical sensitivity of the PCR for the detection of *C. sinensis*, *O. viverrini* and *H. taichui* DNA. The assay's ability to detect artificially mixed infections with varying ratios of *C. sinensis* and *O. viverrini* with *H. taichui* were also assessed.

Assuming microscopy as the ‘gold standard’, the diagnostic sensitivity, and specificity together with their 95% confidence intervals were calculated for the PCR using the Wilson method.

### PCR-linked restriction fragment length polymorphism (RFLP)

The assay's ability to detect artificially mixed infections of *O. viverrini* and *C. sinensis* was assessed by development of a PCR-RFLP as both species produced PCR products that could not be differentiated by size. Amplified ITS-2 products of RTFlukeFa – RTFlukeRa for *C. sinensis* and *O. viverrini* were digested with AcuI (New England Biolabs). According to the restriction profile generated by Nebcutter V2.0 (New England Biolabs), *O. viverrini* does not possess a restriction site for AcuI and remains uncut (375 bp), whereas *C. sinensis* has a single AcuI site and gives rise to two bands at 286 bp and 95 bp. Ten microlitres of PCR product were digested with 2.5 units of the restriction endonuclease AcuI (New England Biolabs) at 37°C for 3 hours in a volume of 20 µl.

## Results

### PCR parameters under experimental conditions

**Figure 1:**
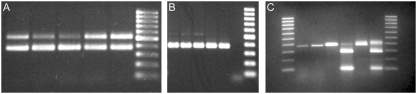
Species identification of *H. taichui*, *O. viverrini* and *C. sinensis* using PCR and PCR-RFLP analysis. Panel A displays PCR products of RTFlukeFa and RTFlukeRa run on a 1.5% agarose gel. From left to right, lanes 1–5 show products of artificially mixed *C. sinensis* (7.6 ng) and *H. taichui* (7.0 ng) DNA in ratios of 1∶1, 2∶1, 3∶1, 1∶2 and 1∶3 respectively, lane 6 shows a 100 base pair DNA ladder. Panel B displays PCR products of RTFlukeFa and RTFlukeRa run on a 1.5% agarose gel. From left to right, lanes 1–5 show products of artificially missed *O. viverrini* (6.0 ng) and *H. taichui* (7.0 ng) DNA in ratios of 1∶1, 1∶2, 1∶3, 2∶1 and 3∶1 respectively, lane 6 displays a negative control and lane 7 shows a 100 base pair DNA ladder. Panel C displays PCR and PCR-RFLP products of RTFlukeFa and RTFlukeRa run on a 2.0% agarose gel. Lanes 1 and 8 display a 100 base pair ladder. Lanes 2 and 3 show amplified products of *O. viverrini* eggs in faeces, undigested and digested products respectively, lanes 4 and 5 show amplified products of *C. sinensis* eggs in faeces, undigested and digested products respectively and lanes 6 and 7 show artificially mixed infections of *O. viverrini* and *C. sinensis* egg-positive faeces undigested and digested products respectively.

Using the primer pair RTFlukeFa and RTFlukeRb, DNA from morphologically identified adults of *O. viverrini*, *C. sinensis* and *H. taichui* gave specific products of 375 bp, 381 bp and 526 bp respectively. The lowest quantity of DNA that could be amplified from individual adults of *O. viverrini*, *C. sinensis* and *H. taichui* was estimated at 0.6 pg, 0.8 pg and 3 pg respectively. Appropriate sized amplicons were produced in reactions artificially mixing DNA of *C. sinensis* and *O. viverrini* separately, with *H. taichui*, in ratios of 1∶1, 1∶2, 1∶3, 3∶1 and 2∶1 (Fig. 1A and 1B). The PCR however, preferentially amplified *O. viverrini* when artificially mixed with *H. taichui*. Weak to negligible bands of *H. taichui* were produced when mixed in ratios of less than 1∶1 with *O. viverrini* (Fig. 1B). The PCR-RFLP patterns for differentiating and detecting mixed infections of *O. viverrini* and *C. sinensis* are displayed (Fig. 1C). The PCR-RFLP was successful at detecting artificially mixed infections of *O. viverrini* and *C. sinensis* in ratios of 1∶1, 1∶2. 1∶3, 3∶1 and 2∶1.

### PCR parameters under field conditions

**Figure 2:**
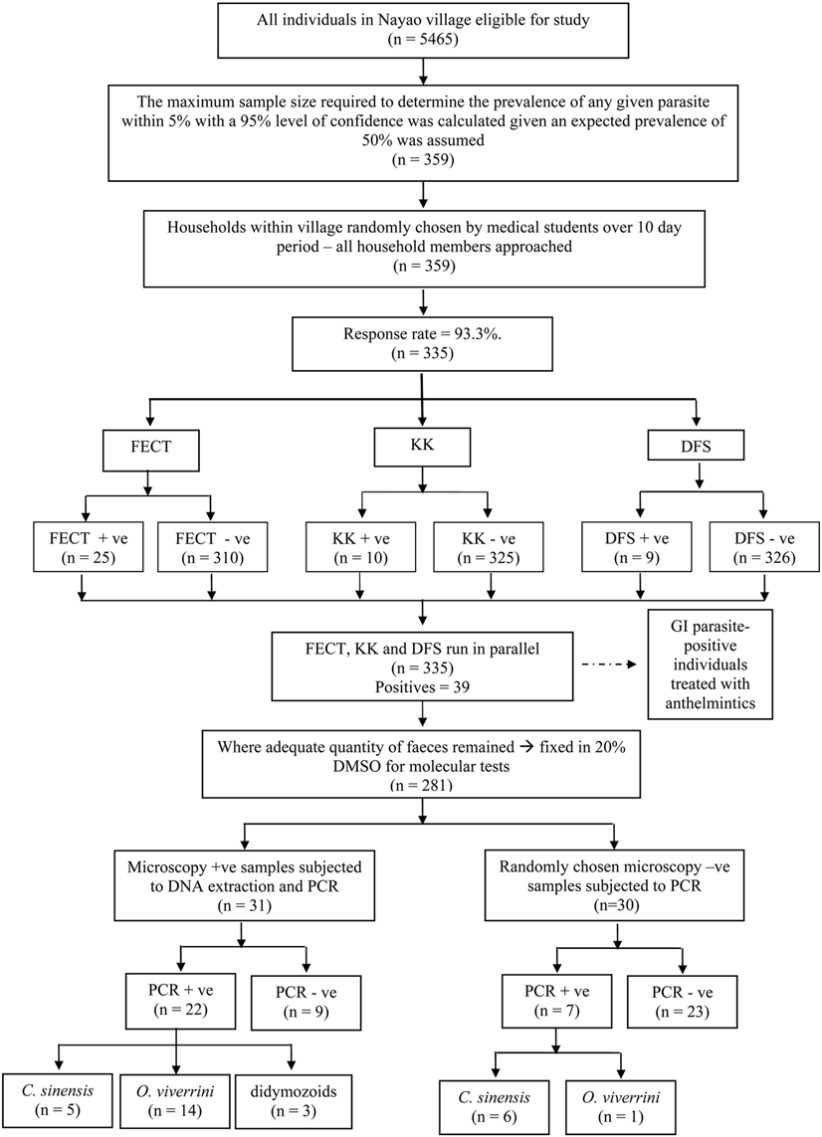
A flow diagram illustrating the study design and summary of diagnostic test results.

For a diagrammatic guide to the study design and summary of diagnostic results refer to Fig. 2. A total of 39 (prevalence 11.6%, 95% CI, 8.6%, 14.92%) individuals were found positive for ‘*Opisthrochis*-like’ eggs in their faeces using a combination of all three microscopic techniques (DFS, KK and FECT). The FECT detected ‘*Opisthorchis*-like’ eggs in more faecal samples (25/31) than the DFS (9/31) and KK (10/31) methods. Using primer pair RTFlukeFa and RTFlukeRb, PCR-positive samples derived from DNA extracted directly from faeces produced a single product corresponding to the expected amplicon size for *O. viverrini* and *C. sinensis* (approximately 380 bp). In three cases, non-specific amplicons were produced in addition to the target PCR product, however these amplicons were too weak (faint) to subject to DNA sequencing. The results of the PCR analysis of 31 microscopy positive and 30 microscopy negative samples are presented in Table 1. The PCR test, when compared to the combined microscopy results yielded a sensitivity of 71.0% (95% CI, 53.4%, 83.9%), and specificity of 76.7% (95% CI, 59.1%, 88.2%). PCR detected an additional seven samples positive for liver fluke that were negative by microscopy. Mixed infections of *O. viverrini* and *C. sinensis* were detected in a single individual by PCR-RFLP.

**Table 1:** Summary of PCR and microscopy results for the detection of liver and intestinal flukes.

	Microscopy positive	Microscopy negative	Total
PCR positive	22	7	29
PCR negative	9	23	32
Total	31	30	61

Morphological and genetic characterisation of the fluke species expelled by the human participant

Adult fluke specimens isolated from a single human participant in the community were identified by morphology as *Clonorchis sinensis* [19]. Two adult fluke specimens subjected to PCR demonstrated 100% DNA sequence homology to the ITS-2 region of *C. sinensis* isolates from Japan and Russia (GenBank accession nos. EF688144 and EF688143).

### Genetic characterisation of fluke species by PCR

**Figure 3:**
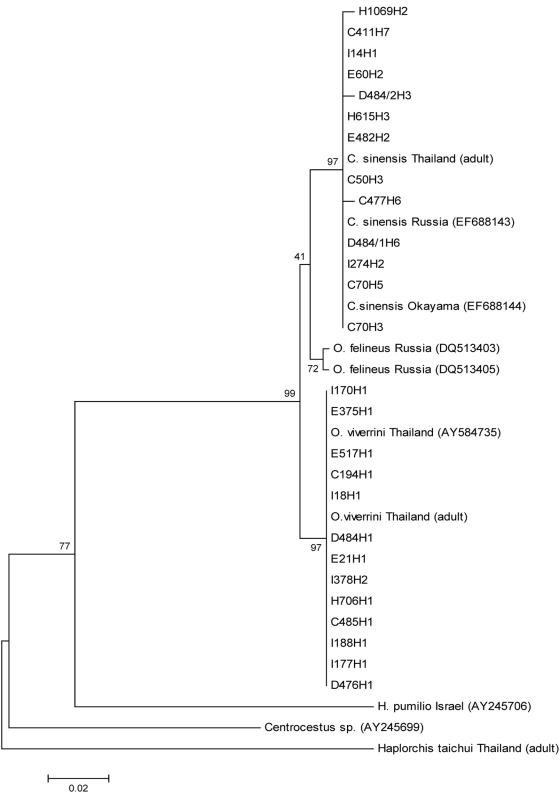
Phenogram construction of the ITS-2 region of the opisthorchid and heterophyid flukes isolated from humans in this study (denoted by their individual code), the adult positive controls and GenBank using the neighbour-joining algorithm and maximum parsimony.

Phenogram construction of the ITS-2 region of the flukes using the neighbour-joining algorithm and maximum parsimony (Fig. 3), produced strong bootstrap support for the placement of 15 PCR-positive samples within a single clade corresponding to *O. viverrini* (GenBank accession number AY584735) and 11 PCR-positive samples corresponding to *C. sinensis* (GenBank accession nos. EF688144, EF688143). Mixed infections with fluke species were not observed by sequencing of the PCR product. Of the 22 individuals found positive for ‘*Opisthorchis*-like’ eggs by both microscopy and by PCR, 14 (64%) were characterised as *O. viverrini* and five (23%) as *C. sinensis*. In addition, three samples (13%) microscopy positive for ‘*Opisthorchis*-like’ eggs produced amplicon sizes of approximately 410 bp each and upon sequencing, were genetically similar to the didymozoids (parasites of fishes), *Rhopalotrema elusiva* (GenBank accession no. AJ224759) and *Indodidymozoon sp.* (GenBank accession no. AJ224754). Six of the microscopy negative but PCR positive samples were genetically characterised as *C. sinensis* and a single sample as *O. viverrini*. No intra-species variation was observed for the *O. viverrini* isolates obtained from this community relative to those obtained from northeast Thailand (positive control and published GenBank isolate AY584735). Apart from three isolates of *C. sinensis* obtained from this community that differed by a transition at a single base, all other isolates of *C. sinensis* were identical to published ITS-2 sequences of *C. sinensis* from Japan (GenBank accession no. EF688144) and Russia (GenBank accession no. EF688143).

## Discussion

A significant finding of this present study was the identification and first report of a community endemic for *C. sinensis* in central Thailand. It is possible that the humans in this community were infected by imported fish or visited *C. sinensis* endemic areas, however when questioned about this, villagers reported only eating fish caught in local ponds or from local village markets and had no history of travelling outside Thailand. Previous studies have only reported *C. sinensis* in Korea, China, Taiwan, Japan, northern Vietnam and the far eastern part of Russia [20]. It is hypothesised that the geographical distribution of clonorchiasis closely parallels the distribution of the snail intermediate host [20], however this assumption may not be as simple as previously thought. Species of *Parafossarulus* and *Bithynia* are most commonly reported to act as first intermediate hosts for *C. sinensis*. The important species in China, Korea and Japan is *Parafossarulus manchouricus* and *P. anamalospiralis* [7]. Other susceptible snails in China are reported as *Bithynia fuchsiana*, *B. longicornis*, *Melanoides tuberculata* and *Assiminea lutea* [20]. In Thailand, *Bithynia siamensis goniomphalos*, *B. s. funiculate* and *B. s. siamensis* act as hosts for *O. viverrini* [19]. In a recent survey of freshwater mollusks in Thailand, an intermediate host of *C. sinensis*, *Melanoides tuberculata* was isolated in the provinces to the south (Chanthaburi) and north (Nakhon Ratchasima Province) of our study area [21]. It is possible that this species of snail may be acting as the natural intermediate host of *C. sinensis* in Thailand. If this is true, then *C. sinensis* may be as geographically widespread as *O. viverrini* in Thailand, reflecting the geographical distribution of *M. tuberculata*, which was isolated from 9/15 districts sampled in the north, east and central regions of Thailand [21]. In saying this however, *M tuberculata* has been shown to harbour both *C. sinensis* and *O. viverrini* in both northern and southern regions of Vietnam, yet surveys to date have found *C. sinensis* to be restricted to the northern provinces and *O. viverrini* to the southern provinces [2]. The distribution of potential snail intermediate hosts therefore does not necessarily reflect the distribution of the liver flukes in Southeast Asia. The PCR test developed in this study provides a useful diagnostic tool for further epidemiological surveys to determine the distribution of these liver flukes in human and intermediate hosts.

The PCR test developed in this study is capable of amplifying *O. viverrini*, *C. sinensis* and potentially the minute intestinal flukes, directly from eggs in faeces. In terms of test parameters, this assay demonstrated a superior sensitivity (Se) to the PCR developed by Stensvold et al. (2006) in the field (Se = 70.9% compared to Se of 45.0%). It also has the added advantage of being able to amplify fluke species other than *O. viverrini*. It may be likely that the presence of faecal inhibitors and/or the unsuccessful ‘cracking open’ of these highly resistant eggs during DNA extraction accounted for the false negative results produced by the PCR in this study. The overall specificity (Sp) of the PCR evaluated using microscopy negative field samples were inferior to those reported by Stensvold et al. (2006) (Sp = 76.7%, compared to Sp: 90.0%), however these assumed ‘false positive’ samples were being compared to the microscopy results (DFS, KK, FECT) which are in themselves not ‘gold standards’. DNA sequences generated from the PCR products of these samples were characterised as either *O. viverrini* or *C. sinensis* and therefore the specificity of this PCR may be under-estimated.

Three faecal samples microscopy positive for ‘*Opisthorchis-like*’ eggs were sequenced and identified as being close to the didymozoids *Rhopalotrema elusiva* and *Indodidymozoon sp*. This is not the first time that eggs of didymozoid flukes have been recovered in human faecal samples [22]. Flukes belonging to the Didymozoidae parasitize a wide range of species of marine fish and ingestion of these adult flukes by humans during the consumption of fish results in the mechanical passage of the relatively thick-shelled eggs into the faeces. Because of their dimensions (35–43×12–28 µm) and morphology (oval, operculate) of the eggs of didymozoid flukes, they can easily be confused with eggs of the Opisthorchiidae, Heterophyidae and Lecithodendriidae. This added confusion may result in further inaccuracies when estimating the prevalence of liver and intestinal flukes in a community using conventional parasitological procedures alone.

The apparent absence of *H. taichui* in the Sanamchaikaet district community was surprising given it is reported commonly in the northeast region of Thailand. It is possible that the PCR failed to amplify eggs from faecal samples with mixed infections of *H. taichui* and *O. viverrini*. Under experimental conditions, the PCR showed good analytical sensitivity for detecting *H. taichui* as a single infection and also when artificially mixed with *C. sinensis*, but failed to amplify a strong band when artificially mixed with *O. viverrini*. Since small intestinal flukes have commonly been found as mixed infections with liver fluke species [5],[23], the PCR developed in this study may not be successful at detecting these infections.

In conclusion, we present data to demonstrate for the first time in Thailand a community endemic for *C. sinensis* infection. This significant finding undoubtedly opens a new chapter for further research into investigating the distribution, and prevalence of *C. sinensis* in Thailand and determining the natural intermediate host species capable of supporting its life cycle. Furthermore, the PCR described herein provides a valuable tool for screening and determining the species of liver and intestinal flukes in epidemiological surveys.

## Supporting Information

Checklist S1 STARD checklist
(0.80 MB JPG)
