# Supplementary figures and images for: A New PCR-Based Approach Indicates the Range of Clonorchis sinensis Now Extends to Central Thailand
Source: PLoS Negl Trop Dis. 2009 Jan 20;3(1):e367. doi: 10.1371/journal.pntd.0000367 (PMC2614470; doi:10.1371/journal.pntd.0000367)

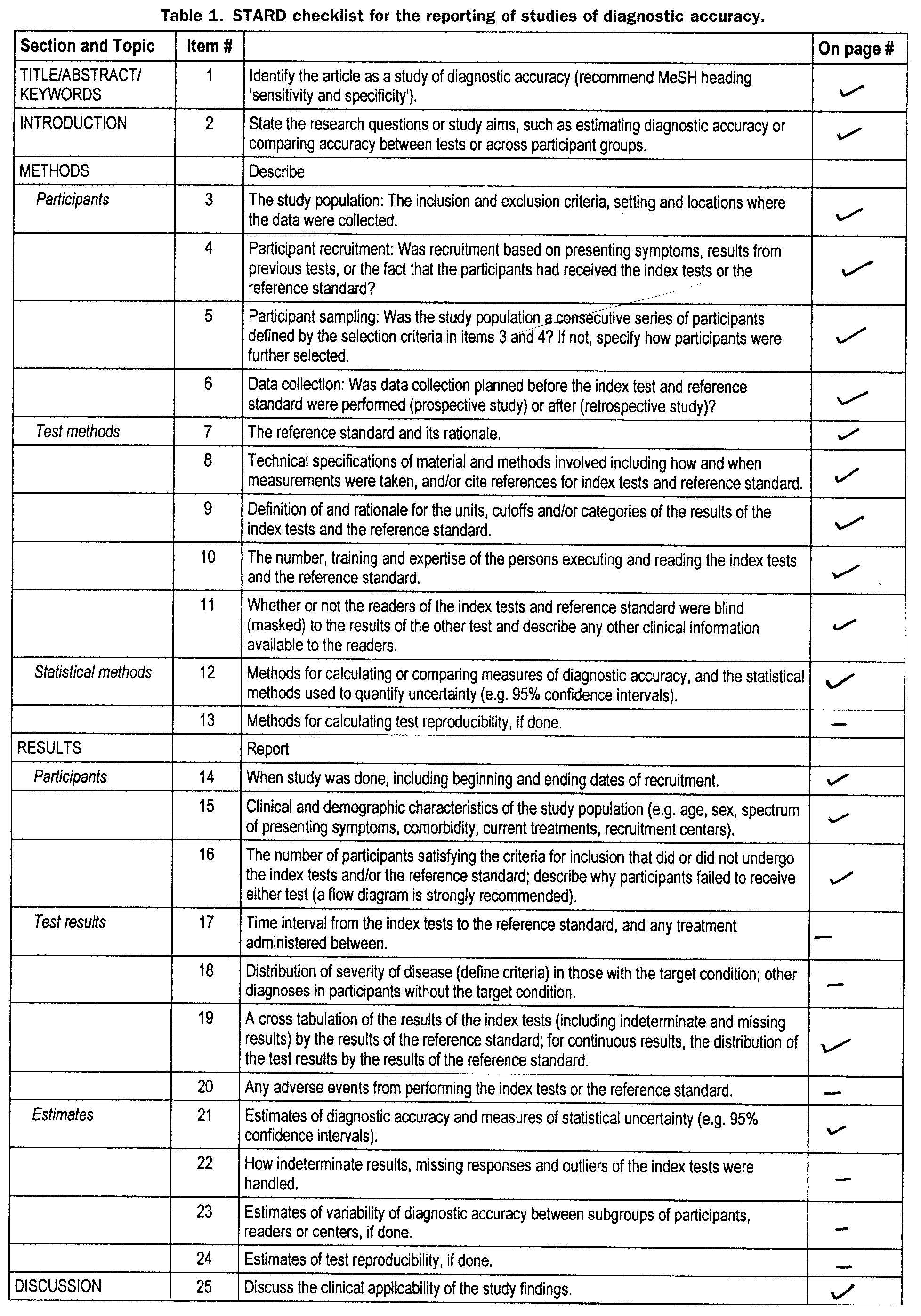

Supplement: Checklist S1 — STARD checklist (0.80 MB JPG) [file pntd.0000367.s001.jpg]
